# The antiinflammatory and electrophysiological effects of fingolimod on penicillin-induced rats

**DOI:** 10.1055/s-0042-1758754

**Published:** 2022-12-29

**Authors:** Canan Akünal Türel, Hümeyra Çelik, İbrahim Ethem Torun, Ayhan Çetinkaya, İdris Türel

**Affiliations:** 1 Bolu Abant İzzet Baysal University School of Medicine, Department of Neurology, Bolu, Turkey.; 2 Bolu Abant İzzet Baysal University School of Medicine, Department of Physiology, Bolu, Turkey.; 3 Bolu Abant İzzet Baysal University School of Medicine, Department of Pharmacology, Bolu, Turkey.

**Keywords:** Rats, Fingolimod Hydrochloride, Anti-Inflammatory Agents, Electrophysiology, Seizures, Epilepsy, Penicillins, Ratos, Cloridrato de Fingolimode, Anti-Inflamatórios, Eletrofisiologia, Convulsões, Epilepsia, Penicilinas

## Abstract

**Background** The fact that inflammation triggers epileptic seizures brings to mind the antiepileptic properties of anti-inflammatory drugs.

**Objective** To investigate the electrophysiological and anti-inflammatory effects of fingolimod on an experimental penicillin-induced acute epileptic seizure model in rats.

**Methods** Thirty-two male Wistar rats were divided into four groups: control (penicillin), positive control (penicillin + diazepam [5 mg/kg]), drug (penicillin + fingolimod [0.3 mg/kg]) and synergy group (penicillin + diazepam + fingolimod). The animals were anesthetized with urethane, and epileptiform activity was induced by intracortical injection of penicillin (500,000 IU). After electrophysiological recording for 125 minutes, IL-1β, TNF-α, and IL-6 were evaluated by ELISA in the serum of sacrificed animals.

**Results** During the experiment, animal deaths occurred in the synergy group due to the synergistic negative chronotropic effect of diazepam and fingolimod. Although not statistically significant, fingolimod caused a slight decrease in spike-wave activity and spike amplitudes in the acute seizure model induced by penicillin ( *p* > 0.05). Fingolimod decreased serum IL-1β ( *p* < 0.05); fingolimod and diazepam together reduced IL-6 ( *p* < 0.05), but no change was observed in serum TNF-α values.

**Conclusion** Even in acute use, the spike-wave and amplitude values of fingolimod decrease with diazepam, anticonvulsant and anti-inflammatory effects of fingolimod will be more prominent in chronic applications and central tissue evaluations. In addition, concomitant use of fingolimod and diazepam is considered to be contraindicated due to the synergistic negative inotropic effect.

## Resumo

**Antecedentes** O fato de a inflamação desencadear crises epilépticas traz à mente as propriedades antiepilépticas dos anti-inflamatórios.

**Objetivo** Investigar os efeitos eletrofisiológicos e anti-inflamatórios do fingolimode em um modelo experimental de crise epiléptica aguda induzida por penicilina em ratos.

**Métodos** Trinta e dois ratos Wistar machos foram divididos em quatro grupos: controle (penicilina), controle positivo (penicilina + diazepam [5 mg/kg]), droga (penicilina + fingolimode [0,3 mg/kg]) e grupo sinergia (penicilina + diazepam + fingolimode). Os animais foram anestesiados com uretano, e a atividade epileptiforme foi induzida por injeção intracortical de penicilina (500.000 UI). Após registro eletrofisiológico por 125 minutos, IL-1β, TNF-α e IL-6 foram avaliados por ELISA no soro dos animais sacrificados.

**Resultados** Durante o experimento, ocorreram mortes de animais no grupo sinérgico devido ao efeito cronotrópico negativo sinérgico do diazepam e do fingolimode. Embora não seja estatisticamente significativo, o fingolimode causou uma ligeira diminuição na atividade pico-onda e nas amplitudes pico no modelo de convulsão aguda induzida pela penicilina ( *p* > 0,05). O fingolimode diminuiu a IL-1β sérica ( *p* < 0,05); fingolimode e diazepam juntos reduziram a IL-6 ( *p* < 0,05), mas não foi observada alteração nos valores séricos de TNF-α.

**Conclusão** Pensa-se que o efeito anticonvulsivante leve de uma dose única de fingolimode será mais proeminente em aplicações crônicas e em avaliações de tecidos centrais. Além disso, o uso concomitante de fingolimode e diazepam é considerado contraindicado devido ao efeito inotrópico negativo sinérgico.

## INTRODUCTION

Epilepsy is a complex disease characterized by different symptoms and findings, neurobiological, cognitive, and psychosocial consequences that progress paroxysmally and seizures are accompanied by different clinics. 1 Even if epilepsy, which is the most common neurological disease in childhood, is symptomatically controlled with antiepileptics, drug resistance over time, 2 and long-term use neurotoxicity 3 have brought up the search for new treatments in epilepsy.

Although the cellular mechanisms of epileptogenesis are not as well-known as the physiology of abnormal discharges during seizures, recently the interest in the role of inflammation in the pathogenesis of epilepsy has been frequently mentioned. 4 During seizures, while intracellular adenosine triphosphate (ATP) decreases, phospholipases activated by the increase of adenosine monophosphate (AMP), adenosine diphosphate (ADP), lactic acid, and calcium cause an increase in free fatty acids, resulting in an increase in inflammatory cytokines (especially IL-1, IL-6 and TNF alpha). Despite seizures causing inflammation, inflammation, in turn, triggers seizures. 5 It has been observed that suppressing inflammation in the treatment of epilepsy significantly alleviates seizures, and studies measuring the efficiency of anti-inflammatory drugs in the treatment of epilepsy are on the agenda. 6 7

Fingolimod is a sphingosine-1 phosphate (S1P) receptor agonist that inhibits the outflow of lymphocytes from the lymph nodes and thymus by acting with an immunomodulatory effect and is widely used in the treatment of multiple sclerosis as an anti-inflammatory agent. 7 8 9 10 Fingolimod, with its lipophilic structure, crosses the blood-brain barrier and affects S1P receptors (astrocyte and microglial cells) located in the central nervous system (CNS), thereby preventing excessive glutamate release. Accordingly, it prevents neurodegenerative diseases with its neuroprotective effect reducing intracellular calcium ion concentration and inflammation. 8 9 10 Fingolimod temporarily creates an antiepileptic period in the genetic Wistar Albino Glaxo Rats from Rijswijk (WAG/Rij) absence epilepsy model, 11 reducing the progression stage, frequency and the number of seizures by reducing neuroinflammation in the pentylenetetrazole-induced kindling model 12 and the lithium-pilocarpine-induced epilepsy. 13 However, its electrophysiological and anti-inflammatory effects within minutes in acute seizures have not been fully clarified.

In the light of this information, the purpose of the present study was to investigate the electrophysiological and anti-inflammatory effects of Fingolimod on an experimental penicillin-induced acute epileptic seizure model in rats.

## METHODS

### Animals

The ethical approval of the study was obtained from the Bolu Abant Izzet Baysal University (BAIBU) Experimental Animals Local Ethics Committee (2021/09).

Two-month-old male Wistar rats, weighing 200-250 g. were obtained from BAIBU Experimental Animals Center and maintained with ad libitum (commercially purchased standard pellet feed) water and pellet feed under 19 ^°^ C ± 2 temperature and 55-60 relative humidity on 12/12 hour light/dark cycle. Thirty-two animals were distributed in 4 groups ( *n* = 8, each): control group (penicillin), positive control (penicillin + diazepam), drug (penicillin + fingolimod), and synergy group (penicillin + fingolimod + diapezam). The animals were caged in group. All experiments were performed between 08:00 and 12:00 a.m.

### Seizure induction and drug applications

**Figure 1:**
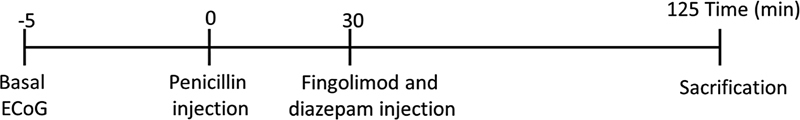
Experimental timeline.

To form an epilepsy model with penicillin, the animals fasted for 24 hours, the upper parts of their heads were shaved under urethane anesthesia (1.25 mg/kg, intraperitoneally [ip]), and the scalps of the animals were fixed on the operating table opened in the rostrocaudal direction, ∼ 3 cm in length with a scalpel. The soft tissue under the left cortex scalp was removed by electrocautery, and the skull bone was removed by making circular movements with a touring motor and by thinning it. After the electrodes were placed and basal activity was recorded for 5 minutes, 500,000 IU penicillin (2.5 µl, icv) 14 was administered to the somatomotor cortex with a Hamilton injector (701N, Hamilton Co., Reno, NV, USA) to induce epileptic activity. The injection coordinates were 2 mm lateral, 1 mm anterior, and 1.2 mm depth of the bregma line. After 30 minutes, saline (0.9%, 0.1 ml, ip, for sham), diazepam (0.1 ml, 5 mg/kg, ip), 15 and fingolimod (0.3 mg/kg ip) 11 were administered to the control, positive control, drug, and synergy groups ( Figure 1 ).

### Electrophysiological assessment

**Figure 2:**
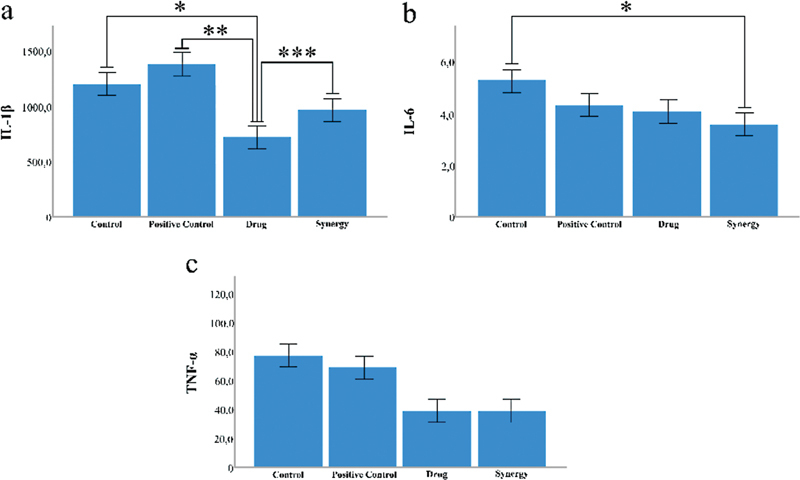
Comparison of proinflammatory cytokines IL-6 (a), IL-1β (b), and TNF-α (c) between groups. Notes: *N* = 8 per group. *: *p* < 0.05.

For electrophysiological recording, two Ag/AgCl ball electrodes were placed with the positive 1 mm anterior to the bregma, 2 mm lateral to the sagittal suture, and the negative 5 mm posterior to the bregma and 2 mm lateral to the sagittal suture. An Ag/AgCl clamp electrode recording gel was applied for grounding and fixed on the right auricle. The activity of electrodes was increased in the BioAmp (PowerLab/8SP, ADInstruments Pty Ltd, Castle Hill, NSW, Australia) interface and instantly transferred to the PowerLab 4/SP (PowerLab/8SP, ADInstruments Pty Ltd, Castle Hill, NSW, Australia) data acquisition unit. Analog signals obtained from the cortex were converted into digital with PowerLab Chart v. 6.0 software package (ADInstruments Pty Ltd., Colorado Springs, CO, United States) and analyzed by transferring them to the computer. After the seizure was formed, 125 minutes were recorded for ( Figures 1 and 2 ).

### Blood collection and biochemical parameters

After electrophysiological recordings were made for all rats, 5 ml intracardiac blood was taken. The blood samples were centrifuged at 4,000 rpm for 10 minutes and stored in eppendorf tubes at - 80 ^°^ C until the biochemical parameters were studied.

### Elisa

Serum levels of IL-1β, IL-6 and TNF-α were determined by using commercially available enzyme-linked immunosorbent assay (ELISA) kits (BT LAB Bioassay Technology Laboratory TNF-α cat no: E0764Ra, IL-6 cat no: E0135Ra, IL-1β cat no: E0119Ra, Zhejiang, China) according to the instructions of the manufacturer.

### Statistical analysis

Data were evaluated in the statistical package program IBM SPSS Statistics for Windows version 25.0 (IBM Corp., Armonk, New York, USA). Descriptive statistics were given as number of units ( *n* ), mean ± standard deviation (x ± SD) (in normally distributed data) and median (Q1-Q3) values (in non-normally distributed data). The normal distribution of the data of numerical variables was evaluated with the Shapiro-Wilk test of normality and Q-Q graphs. The homogeneity of the data was checked with the Levene test for equality of variances. Comparisons between groups were made with one-way analysis of variance (ANOVA) or Kruskal-Wallis analysis for non-normal distributed variables. Tukey HSD was used for normally distributed variables and the Bonferroni corrected Mann-Whitney U test was used for non-normally distributed variables as post-doc test. P-values < 0.05 were considered statistically significant.

## RESULTS

In the inflammatory markers studied in serum samples, IL-1β was found to be significantly higher in the control, positive control, and synergy groups compared with the drug group ( *p* < 0.05). IL-6 was found to be significantly higher in the control group than in the synergy group ( *p* < 0.05). No statistically significant change was found in TNF-α values ( *p* > 0.05) ( Figure 2 ).

**Figure 3:**
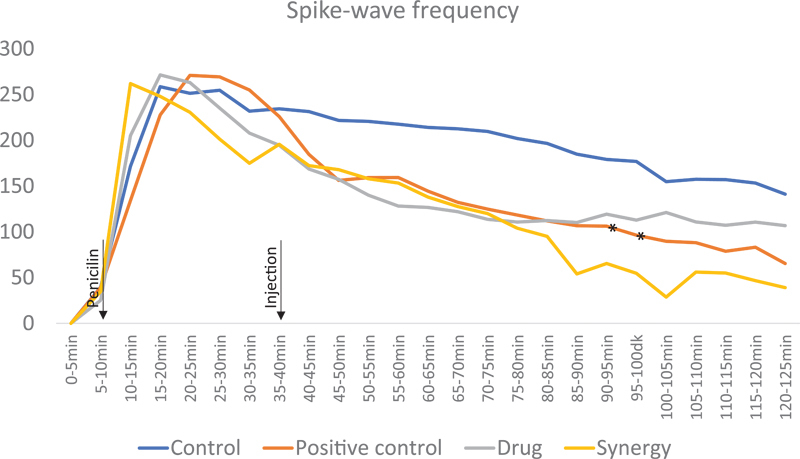
Measurement of spike-wave frequencies among groups during 125 minutes. Note: *means there was statistical significance between positive control and drug groups (p < 0.05).

When the electrophysiological results were evaluated, the mean spike-wave activity values measured in the first 5 minutes of epileptic activity were similarly not significant in any group ( *p* < 0.05). There was statistical significance in the median values of spike-wave activity throughout the EEG recording in 91-95, 96-100, 101-105, and in106-110 minutes between positive control and control groups ( *p* < 0.05); and in 91-95 and 96-100 minutes between positive control and drug groups ( *p* < 0.05) ( Table 1 , Figure 3 ).

**Table 1:** The values of spike-wave frequency activity and amplitude measured between the 0 ^th^ and 125 ^th^ minute of electrophysiological recording

Time (minute)	Control		Positive control		Drug		Synergy		*p-value*	
	Spike-wave	Amplitude (mV)	Spike-wave	Amplitude (mV)	Spike-wave	Amplitude (mV)	Spike-wave	Amplitude (mV)	Spike-wave	Amplitude
0-5	0.00 ± 0	0.9212 ± 0.39	0.00 ± 0	0.7780 ± 0.27	0.00 ± 0	0.7734 ± 0.23	0.00 ± 0	1.1475 ± 0.46	0.00	0.771
11-15	182	3,0789	150.5	1,3893	184.5	2,5018	273	2,0799	0.469	0.646
21-25	257.5	4,02	268.5	2,09	260.5	3,11	222	2,92	0.139	0.845
31-35	231.87	3,7933	255.00	2,9262	207.87	2,6385	201,85	3,1629	0.138	0.359
41-45	248.5	3,7022	180	2,6096	172.5	2,5740	208	1,8931	0.884	0.16
51-55	223.5	2,9345	160.5	2,3046	159	2,7325	199	1,8182	0.572	0.046 ^*^
61-65	210	2,8598	146.5	1,9090	152.5	2,6792	186	1,7652	0.129	0.043 ^*^
71-75	225.5	2,4224	132.5	1,7888	137.5	2,7338	157	1,6589	0.095	0.084
81-85	238	2,5119	103.5	1,7534	126	2,2570	134	1,5201	0.060	0.024 ^*^
91-95	201.5	2,0382	90.5	1,7764	135	2,3607	0	0,5845	0.038 ^*^	0.073
101-105	176.5	1,963	70	1,2092	129.5	2,0567	0	0,3788	0.024 ^*^	0.058
111-115	160	1,719	69	1,2463	121.5	2,0821	0	0,5268	0.061	0.025 ^*^
121-125	120	1,5006	62.5	1,2833	114	1,7271	0	0,2534	0.052	0.015 ^*^

Notes: The mean values and standard deviations of the normally distributed variables, and the median values of the non-normally distributed variables are given.

*: there was statistical significance between groups ( *p* < 0.05).

Measurement of spike-wave frequencies among groups during 125 min. * means there was statistical significance between positive control and drug groups (p < 0.05)

**Figure 4:**
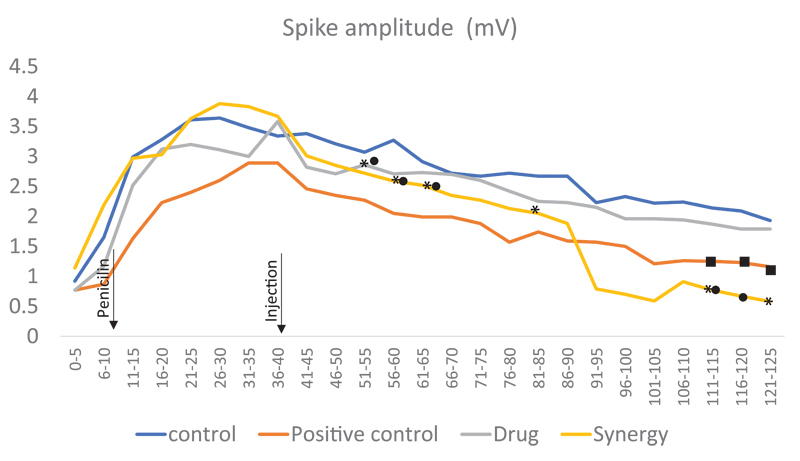
Spike amplitude graph between groups during 125 min of measurement. Spike amplitude grapfh between groups during 125 minutes of measurement. Note: *means there was statistical significance between synergy and control groups ( *p* < 0.05). :means there was statistical significance between the synergy and drug groups ( *p* < 0.05). :there was statistical significance between the drug and control groups ( *p* < 0.05).

Five animals in the synergy group died in the 45 ^th^ minute during EEG recording, before completing the experiment. The tissues of the animals were taken immediately. Only 3 animals completed the whole spike-amplitude measurements taken for 2 hours. In 51-55, 56-60, 61-65, 81-85, 111-115, and 121-125 minutes between synergy and the control groups, there was a statistically significant decrease in mV ( *p* < 0.05) ( Table 1 , Figure 4 ). Also, there was statistical significance in the median values of spike-amplitude values throughout the entire EEG recording in 51-55, 56-60, 61-65, 111-115 and 116-120 minutes when synergy was compared to the drug groups ( *p* < 0.05). There was statistical significance between positive control and control groups in 81-85, 111-115, 116-120, and 121-125 minutes. It was observed that the drug group was not as effective as the positive control with statistical significance in 111-115, 116-120, and 121-125 minutes. A decrease in seizure activity was detected, probably because they were pre-ex.

## DISCUSSION

Our study is the first in the literature to show the electrophysiological and anti-inflammatory effects of fingolimod in rats with penicillin-induced acute seizures. Fingolimod was not as effective against spike-wave as diazepam, but it was found to be effective in reducing the spike-wave values of acute crisis numerically. The combined use of diazepam and fingolimod was found to be more beneficial in reducing spike-amplitude values than the use of fingolimod alone. Fingolimod decreased serum IL-1β; and fingolimod and diazepam together reduced IL-6, but no change in serum TNF-α values was observed.

Inflammatory mediators released in neuroinflammation lower the seizure threshold 16 by upregulating gene expression of neuronal cell death and synaptic plasticity. 17 Also, it has been shown that inflammatory cytokines such as IL-1β, IL-6, TNF-α, and TGF-β 18 are high due to glial activity in epilepsy, 19 and suppressing these factors has a healing effect on seizures. 20 It is known that astrocytes are activated in epilepsy, and proinflammatory substances such as cytokines, enzymes, and adhesion molecules secreted by activated astrocytes 21 rectify K+ channels inward and impair potassium ion uptake decreasing the expression of glutamine synthase, glutamate dehydrogenase and glial gamma-aminobutyric acid (GABA) transporter. 22 23 Collectively, these events increase neuronal hyperexcitability, leading to the development of epilepsy.

Fingolimod reduces neuronal hyperexcitability 13 based on its anti-neuroinflammatory characteristic, antigliotic effect, and neuromyelin protection. 24 In the chronic epilepsy model induced by lithium-pilocarpine, fingolimod has been shown to improve the incidence, duration, frequency and severity of spontaneous convulsions by reducing neuronal loss in the hippocampus, microglia and astrocyte activation, and hippocampal TNF-α and IL-1β expression. 13 In the pentylenetetrazole-induced neonatal chronic epilepsy model, fingolimod prevented long-term cognitive disorders of epilepsy by improving seizure severity by reducing hippocampal TNF-α through inflammation. 2 In our study, fingolimod decreased serum IL-1β in accordance with the literature, but a nonstatistical decrease was observed in TNF-α. In this case, the results of studies 13 25 showing the protective effect of fingolimod on seizures may have caused it to be seen more sharply than our study: chronic epilepsy model, fingolimod dose (1 mg/kg, adult, 0.3 mg/kg, neonatal), the type of central tissue. It is understood that the anti-inflammatory and antiepileptic effects of fingolimod may be more pronounced in chronic drug applications. In addition, it was observed that serum IL-6 was significantly lower in our synergy group than in the other groups. It is mentioned in the literature that diazepam has anti-inflammatory effects. 26 Therefore, fingolimod and diazepam may have decreased IL-6 more than fingolimod alone, possibly with a synergistic effect.

There are very few studies in the literature in which the effects of fingolimod in epilepsy are evaluated electrophysiologically. Although acute and subacute doses (0.3 and 1 mg/kg) of fingolimod did not affect absence seizures in EEG in the genetically induced WAG/Rij absence epilepsy model, it was observed that low-dose reduced spike-wave activity duration in absence seizures in long-term early treatment. However, 5 months after the treatment was stopped, the duration of the seizures was at the same level as the controls 11 ; the research did not give EEG data in minutes. Although the seizure style was different, it was found that fingolimod was not effective in acute seizures, similar to our results. In another study, in kindling epilepsy model induced by pentylenetetrazole, it was observed that two different doses of fingolimod (0.3 and 1 mg/kg) improved myelin damage in the hippocampus by reducing microglial and astrocytic activity, and decreased the stage of seizures with an anti-inflammatory effect. 12 Although the EEG recordings of the study were given visually, it was determined that 0.3 mg/kg fingolimod was more successful in reducing the frequency of seizures in chronic administration. At this stage, long-term use of low-dose fingolimod affects seizures in a way that is reflected in the EEG. It could be said that the anti-inflammatory effects of high-dose fingolimod are more dominant than the electrophysiological effect in epilepsy.

Diazepam, from the benzodiazepine group, is frequently used as a positive control group in experimental epilepsy studies, and it is considered as a good choice to compare the benefits of the target molecule. 1 In our study, the effects of fingolimod were compared with diazepam, and most of the animals in the synergistic effect group, in which fingolimod and diazepam were administered together, died after ∼ 45 minutes and could not complete the experiment. There is no other study in the literature in which the two drugs were used together. Both fingolimod 27 and diazepam 28 are known to have negative chronotropic effects on the heart. We speculate that the animals probably entered bradycardia with a synergistic effect and died suddenly from cardiac causes.

Our limitations are the lack of different fingolimod doses and cytokines were not evaluated in a central tissue together with the serum. Our original aspect is that the study is the first to evaluate the electrophysiological effects of fingolimod in acute seizures with minute interaction. In conclusion, when our study results and the literature are evaluated together, although long-term use of fingolimod showed the anti-inflammatory effect in epilepsy more prominently, it caused a decrease in serum IL-1β even in acute application. The curative effect of fingolimod for pathophysiology, not symptomatic, suggests that it may be a good alternative or supplement molecule to antiepileptics in the future.
